# The Extent of mRNA Editing Is Limited in Chicken Liver and Adipose, but Impacted by Tissular Context, Genotype, Age, and Feeding as Exemplified with a Conserved Edited Site in COG3

**DOI:** 10.1534/g3.115.022251

**Published:** 2015-12-02

**Authors:** Pierre-François Roux, Laure Frésard, Morgane Boutin, Sophie Leroux, Christophe Klopp, Anis Djari, Diane Esquerré, Pascal GP Martin, Tatiana Zerjal, David Gourichon, Frédérique Pitel, Sandrine Lagarrigue

**Affiliations:** * Agrocampus Ouest, Unité Mixte de Recherche 1348 Physiologie, Environnement et Génétique pour l’Animal et les Systèmes d’Élevage, F-35000 Rennes, France; † Institut National de la Recherche Agronomique, Unité Mixte de Recherche 1348 Physiologie, Environnement et Génétique pour l’Animal et les Systèmes d’Élevage, F-35000 Rennes, France; ‡ Unité Mixte de Recherche Institut National de la Recherche Agronomique, Génétique, Physiologie et Systèmes d’élevage, Institut National de la Recherche Agronomique, F-31326 Castanet Tolosan, France; § École Nationale Supérieure Agronomique de Toulouse, Génétique, Physiologie et Systèmes d’élevage, Institut National de la Recherche Agronomique, F-31326 Castanet Tolosan, France; ** École Nationale Vétérinaire de Toulouse, Génétique, Physiologie et Systèmes d’élevage, Institut National de la Recherche Agronomique, F-31326 Castanet Tolosan, France; †† Institut National de la Recherche Agronomique, Sigenae Unité de Recherche 875 Biométrie et Intelligence Artificielle, F-31326 Castanet-Tolosan, France; ‡‡ Institut National de la Recherche Agronomique, GeT-PlaGe Genotoul, F-31326 Castanet-Tolosan, France; §§ Institut National de la Recherche Agronomique, Unité Mixte de Recherche 1331 Research Center in Food Toxicology, F-31027 Toulouse, France; *** Institut National de la Recherche Agronomique, Unité Mixte de Recherche 1313 Génétique Animale et Biologie Intégrative, F-78350 Jouy-en-Josas, France; ††† Institut National de la Recherche Agronomique, Pôle d’Expérimentation Avicole de Tours, F-37380 Nouzilly, France

**Keywords:** mRNA editing, chicken, RNA-seq, DNA-seq, liver and adipose tissue

## Abstract

RNA editing is a posttranscriptional process leading to differences between genomic DNA and transcript sequences, potentially enhancing transcriptome diversity. With recent advances in high-throughput sequencing, many efforts have been made to describe mRNA editing at the transcriptome scale, especially in mammals, yielding contradictory conclusions regarding the extent of this phenomenon. We show, by detailed description of the 25 studies focusing so far on mRNA editing at the whole-transcriptome scale, that systematic sequencing artifacts are considered in most studies whereas biological replication is often neglected and multi-alignment not properly evaluated, which ultimately impairs the legitimacy of results. We recently developed a rigorous strategy to identify mRNA editing using mRNA and genomic DNA sequencing, taking into account sequencing and mapping artifacts, and biological replicates. We applied this method to screen for mRNA editing in liver and white adipose tissue from eight chickens and confirm the small extent of mRNA recoding in this species. Among the 25 unique edited sites identified, three events were previously described in mammals, attesting that this phenomenon is conserved throughout evolution. Deeper investigations on five sites revealed the impact of tissular context, genotype, age, feeding conditions, and sex on mRNA editing levels. More specifically, this analysis highlighted that the editing level at the site located on *COG3* was strongly regulated by four of these factors. By comprehensively characterizing the mRNA editing landscape in chickens, our results highlight how this phenomenon is limited and suggest regulation of editing levels by various genetic and environmental factors.

RNA editing has become a generic term for a wide array of post-transcriptional processes that change the mature RNA sequence relative to the corresponding encoding genomic DNA matrix. This phenomenon, which is almost limited to eukaryotes with some exceptions, is characterized by nucleotide insertion, deletion, or substitution in various types of RNAs including mRNAs (Knoop 2011), tRNAs (Börner *et al.* 1996; Gott *et al.* 2010; Torres *et al.* 2014), miRNAs (Warnefors *et al.* 2014), and rRNAs (Eifler *et al.* 2013; Valach *et al.* 2014), and is likely to contribute to RNA diversity. Until recently, this mechanism was considered relatively rare in vertebrates, mainly restricted to brain-specific substrates and repetitive regions of the genome (Bass 2002), and limited to extensively validated ADAR-mediated adenosine to inosine (A-to-I) substitutions and APOBEC-mediated cytosine to uracil (C-to-U) changes (Knoop 2011).

Since 2009, the advent of high-throughput sequencing technologies has enabled the study of this phenomenon at a transcriptome-wide scale and progressively challenged this view, with estimates ranging from several hundred (Ju *et al.* 2011; Kleinman *et al.* 2012) to several thousand (Clop *et al.* 2006; Li *et al.* 2011; Bahn *et al.* 2011; Peng *et al.* 2012; Ramaswami *et al.* 2012; Park *et al.* 2012; Chen 2013; Kang *et al.* 2015), and even millions of (Bazak *et al.* 2014) mRNA edited sites throughout mammalian genomes. According to some of these mRNA editing screening studies, mRNA recoding is an extremely common process that greatly contributes to transcript diversity. Furthermore, most of these studies report mRNA editing events leading to transversions that cannot be explained in the light of our current knowledge regarding the molecular bases of mRNA recoding (Li *et al.* 2011; Ju *et al.* 2011; Bahn *et al.* 2011; Peng *et al.* 2012; Chen 2013; Kang *et al.* 2015), suggesting the existence of currently uncharacterized mRNA editing mechanisms and novel molecular components implied in gene expression regulation. The conclusions raised by these studies regarding the extent and nature of mRNA recoding, if further supported, would deeply impact our understanding of gene expression regulation and transcriptional modification.

Facing contradictory results regarding the extent of mRNA editing, a large number of studies and comments have pointed to the requirement for comprehensive and rigorous bioinformatics pipelines to limit technical artifacts in editome characterization (Schrider *et al.* 2011; Kleinman and Majewski 2012; Lin *et al.* 2012; Pickrell *et al.* 2012; Kleinman *et al.* 2012; Piskol *et al.* 2013; Lagarrigue *et al.* 2013). Working with short-read sequencing data for the detection of polymorphisms requires careful dealing with technical artifacts related to mapping on paralogous or repetitive regions (Malhis and Jones 2010; Treangen and Salzberg 2012), mapping errors at splice sites (Park *et al.* 2012), or systematic and random sequencing errors (Nakamura *et al.* 2011; Meacham *et al.* 2011). This is especially the case when screening for mRNA editing events, since all of these artifacts are likely to generate artificial discrepancies between genomic DNA and mRNA further interpreted as edited sites. In this context, the huge variation regarding the extent of intratissue and intraspecies mRNA editing revealed in the literature could be in part due to the varying level of stringency of bioinformatics filters used to control these error prone artifacts, and whether biological replication is considered or not.

As shown in Table 1, most of the 25 RNA-seq-based mRNA editing screening studies performed on vertebrates have not considered matched genomic DNA sequences to detect mRNA recoding, but rather have considered either a consensus genomic sequence for the species studied, or expressed sequence tag (EST) databases to remove false positives arising from potential genomic polymorphisms, therefore occulting unreferenced individual variations (Bahn *et al.* 2011; Danecek *et al.* 2012; Gu *et al.* 2012; Ramaswami *et al.* 2012; Cattenoz *et al.* 2013; Lagarrigue *et al.* 2013; Chen 2013; Bazak *et al.* 2014; Blanc *et al.* 2014; Toung *et al.* 2014; Sakurai *et al.* 2014; Chan *et al.* 2014; Zhang and Xiao 2015). More strikingly, while it is fully acknowledged that filtering on minor allele frequency is required to select high-quality genomic polymorphisms (The 1000 Genomes Project Consortium 2010, 2012), some mRNA editing screening studies still consider that reproducibility across biological replicates is not a mandatory criterion for considering a difference between DNA and RNA as a reliable editing event (Picardi *et al.* 2012; Peng *et al.* 2012; Ramaswami *et al.* 2012; Kleinman *et al.* 2012; Park *et al.* 2012; Cattenoz *et al.* 2013; Chen 2013; Bazak *et al.* 2014; Sakurai *et al.* 2014; Chen *et al.* 2014; Chan *et al.* 2014; Kang *et al.* 2015; Zhang and Xiao 2015). However, as depicted in Figure 1, considering biological replication clearly affects the total number of editing events detected in high-throughput-based screening studies, since the number of differences between DNA and RNA reported appears to be directly negatively correlated with the number of biological replicates considered. From a methodological point of view, this study proposes a rigorous strategy to identify mRNA editing using both mRNA and genomic DNA high-throughput sequencing, taking into account sequencing and mapping artifacts, as well as biological replicates, to control the false positive rate. The efficiency of this approach has already been validated in our previous study on chicken embryo mRNA editing (Frésard *et al.* 2015). To strictly control multimapping, we looked for mRNA sequences spanning edited sites in unmapped genomic DNA sequences, allowing the consideration of potential errors and gaps in the reference assembly that still represent roughly 15% of the chicken genome (Wicker *et al.* 2005; Schmid *et al.* 2015).

**Table 1:** Whole transcriptome mRNA editing screening studies in vertebrates

Study	Species	Cells	Edited Sites (N)	Matched DNA*^a^*	Replicates*^b^*	Potential Biases
(Ju *et al.* 2011)	*H. sapiens*	Immortalized B cells	1809	Yes	2/17	Splice, homopolymer, strand, extremity
(Li *et al.* 2011)	*H. sapiens*	Immortalized B cells	28,766	Yes	2/27	Splice, homopolymer, multimapping, strand, extremity
(Bahn *et al.* 2011)	*H. sapiens*	Glioblastoma cells	10,000	No	2/2	Splice, homopolymer, multimapping, strand, extremity
(Peng *et al.* 2012)	*H. sapiens*	Immortalized B cells	22,688	Yes	1/1	Splice, homopolymer, extremity
(Park *et al.* 2012)	*H. sapiens*	14 ENCODE cell lines	5695	No	1/1	Homopolymer, extremity, multimapping
(Kleinman *et al.* 2012)	*H. sapiens*	Immortalized B cells	1503	Yes	1/2	Homopolymer
(Ramaswami *et al.* 2012)	*H. sapiens*	ENCODE cell lines	150,865	No	1/2	Strand, multimapping
(Picardi *et al.* 2012)	*H. sapiens*	Spinal cord cells	15	Yes (Exome)	1/1	Homopolymer, multimapping, strand
(Bazak *et al.* 2014)	*H. sapiens*	16 tissues	1,586,270	No	1/1	Homopolymer, multimapping, strand
(Chen 2013)	*H. sapiens*	7 ENCODE cell lines	259,385	No	1/2	Multimapping
(Chan *et al.* 2014)	*H. sapiens*	Liver cells	20,007	No	1/3	Splice, homopolymer, multimapping, strand, extremity
(Mo *et al.* 2014)	*H. sapiens*	Prostate cancer cells	16,194	Yes	2/10	Homopolymer, multimapping, strand, extremity
(Sakurai *et al.* 2014)	*H. sapiens*	Brain cells	19,791	No	1/1	Splice, homopolymer, multimapping, strand, extremity
(Toung *et al.* 2014)	*H. sapiens*	Immortalized B cells	5997	No	2/2	Multimapping, strand, extremity
(Zhang and Xiao 2015)	*H. sapiens*	Immortalized B cells	22,715	No	1/1	Multimapping, extremity
(Hu *et al.* 2015)	*H. sapiens*	Hepatocellular carcinoma cells	900	Yes	6/6	Splice, multimapping, strand
(Kang *et al.* 2015)	*H. sapiens*	Liver cells	485,684	Yes	1/9	Splice, multimapping, strand
(Danecek *et al.* 2012)	*M. musculus*	Brain cells	7389	No	2/2	Homopolymer, multimapping
(Gu *et al.* 2012)	*M. musculus*	Liver, adipose, and bone cells	253	No	3/3	Homopolymer, multimapping, extremity
(Lagarrigue *et al.* 2013)	*M. musculus*	Liver and adipose cells	63 and 188	No	4/6	Multimapping
(Cattenoz *et al.* 2013)	*M. musculus*	Brain cells	665	No	1/1	Splice, homopolymer, multimapping, strand, extremity
(Blanc *et al.* 2014)	*M. musculus*	Intestine and liver cells	500	No	1/1	Homopolymer, multimapping, extremity
(Chen *et al.* 2014)	*R. macaque*	Prefrontal cortex, cerebellum, muscle, kidney, heart, testis, and lung cells	31,250	Yes	1/1	Homopolymer, multimapping, extremity
Frésard *et al.* 2015	*G. gallus*	Whole embryo	40	Yes	2/8	—
Roux *et al.* (The present study)	*G. gallus*	Liver and adipose cells	11 and 17	Yes	3/8	—

a If “Yes”: individual genomic DNA information is used to account for potential private individual genomic polymorphisms. If “No”: potential private genomic polymorphisms are defined considering either genomic variant databases such as dbSNPs, or strain-specific consensus genomic sequence in the case of studies based on clonal mouse strains.

b Ratio between the number of biological replicates considered for reporting a candidate difference between DNA and mRNA as a true mRNA editing event and the total number of biological replicates available in the study for a given cell type.

**Figure 1:**
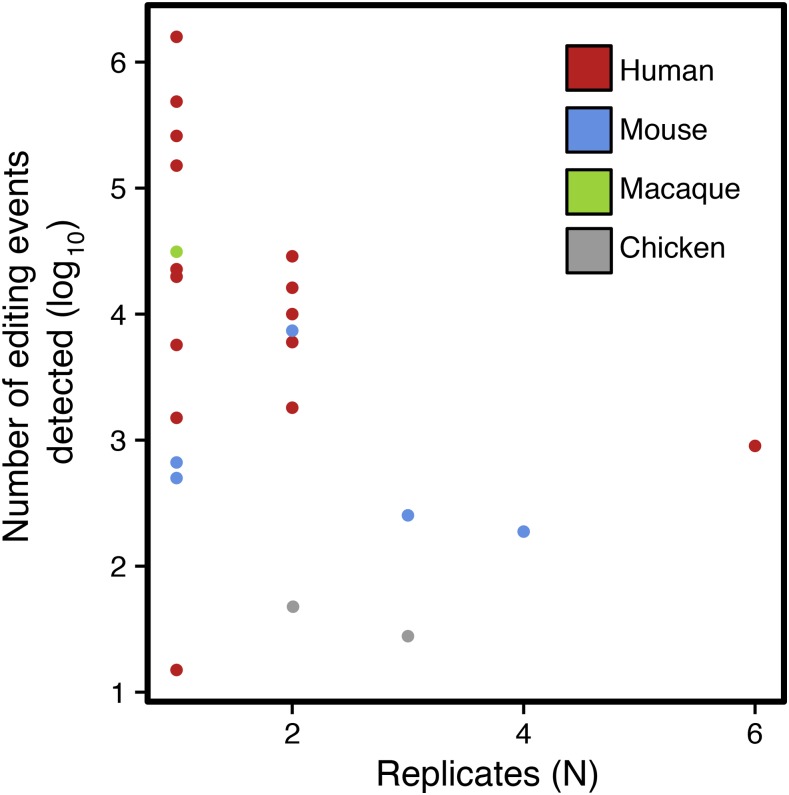
mRNA editing screening studies based on high-throughput sequencing in the literature. This graph describes the numbers of mRNA editing events detected (log_10_) across RNA-seq-based screening studies as a function of the numbers of biological replicates considered to declare an event as a true positive.

From a biological perspective, in addition to our recent work screening mRNA editing in chicken whole embryos (Frésard *et al.* 2015), this study answers the evident lack of transcriptome-wide mRNA editing screening investigations focusing on nonmammalian vertebrates such as birds, that could contribute to our understanding of the evolutionary basis of RNA editing. Indeed, as depicted in Table 1, except for our works, all mRNA editing screening studies in vertebrates to date have focused on primates or mouse transcriptomes. At present, the origins of RNA editing are still rather obscure and, even though it is proposed that RNA editing may have arisen several times in different phyla throughout evolution, it remains unclear whether selection was involved or not (Gommans *et al.* 2009; Gray *et al.* 2010; Gray 2012). While chickens are extensively used as a model organism in developmental biology (Davey and Tickle 2007), they also bridge the evolutionary gap between mammals and other vertebrates. Therefore, they stand as an ideal species to explore the conservation of mRNA editing events in vertebrates throughout evolution. In addition, our knowledge related to the regulation of mRNA editing levels and factors enhancing or repressing mRNA recoding is still limited. Hitherto, few studies have been carried out to assess whether the genetic background, sex, feeding conditions, or age influence levels of mRNA recoding. Most of these studies have targeted the extensively studied APOBEC-mediated C-to-U editing event that occurs in mammalian *APOBEC1* mRNA, revealing the influence of ethanol intake (Lau *et al.* 1995; Van Mater *et al.* 1998), insulin (von Wronski *et al.* 1998), obesity (Phung *et al.* 1996), and diet (Funahashi *et al.* 1995) on *APOBEC1* mRNA editing levels. Others have focused on previously described ADAR-mediated editing events in primate (Li *et al.* 2013), mouse (Gan *et al.* 2006), or rat (Holmes *et al.* 2013) transcriptomes, highlighting an insulin-dependent activity of ADAR in mouse pancreas (Gan *et al.* 2006), or suggesting the influence of aging on ADAR-mediated mRNA editing in human, mouse, and pig (Wahlstedt *et al.* 2009; Shtrichman *et al.* 2012; Venø *et al.* 2012). Better characterization of environmental and genetic factors influencing the level of mRNA recoding would offer new insights on the role of mRNA editing in vertebrates.

In this study, we report results from the first genome-wide characterization of chicken liver and adipose mRNA editomes, based on both genomic DNA and mRNA high-throughput sequencing. Our results confirm the low extent of mRNA recoding in chicken and the absence of non A-to-I editing events in this species, in agreement with what has already been shown for chicken embryos (Frésard *et al.* 2015). We also highlight that the mRNA editing level is impacted by genetic and environmental factors such as tissular context, genotype, age, and, to a minor extent, by feeding conditions and sex. As exemplified with the recoding event located on *COG3* and confirmed at other positions, the mRNA editing level is tightly dependent on several environmental and genetics factors.

## Materials and Methods

### Ethics statement

Chickens were bred at INRA, UE1295 Pôle d’Expérimentation Avicole de Tours, F-37380 Nouzilly, in accordance with European Union Guidelines for animal care, following the Council Directives 98/58/EC and 86/609/EEC. Animals were maintained under standard breeding conditions and subjected to minimal disturbance. The farm is registered with the French Ministry of Agriculture under license number C37–175–1 for animal experimentation. The experiment was performed under authorization 37–002 delivered to D. Gourichon.

### Tissue collection and library preparation

Two experimental meat-type chicken lines were divergently selected for seven generations using the ratio between abdominal fat weight and whole animal weight at 9 wk as a fattening index, while maintaining the live body weight constant (Leclercq *et al.* 1980). After selection, the two lines were maintained by carefully limiting inbreeding. Four nine-week-old males from the 35^th^ generation in each line were slaughtered by electronarcosis and immediate bleeding. Liver and abdominal adipose tissue were then harvested and stored in nitrogen. Liver genomic DNA and total liver and adipose RNA were concurrently extracted according to the manufacturer’s instructions using the AllPrep DNA/RNA Mini Kit (Agilent, Agilent Technologies, Santa Clara, CA). RNA quality was assessed on a BioAnalyzer 1000 (Agilent Technologies, Santa Clara, CA) and RIN (RNA Integrity Number) ≥9 were required.

### Sequencing

#### RNA sequencing:

Libraries with a mean insert size of 200 bp were prepared according to the manufacturer’s instructions for RNA-seq library preparation, selecting polyadenylated mRNA using the TruSeq RNA Sample Prep Kit (Illumina, San Diego, CA) from each sample. Samples were tagged using a barcode sequence for subsequent identification, amplified by PCR and quantified by qPCR using the QPCR Library Quantification Kit (Agilent Technologies, Santa Clara, CA). A total of 16 libraries were sequenced in paired-ends 2 × 101 bp in triplicate on three different lanes on the Illumina HiSeq 2000 sequencer using the TruSeq PE Cluster Kit v3 (Illumina, San Diego, CA), the cBot SBS Kit v3 (Illumina, San Diego, CA) and the TruSeq SBS Kit v3 (Illumina, San Diego, CA). After quality checks and adapter trimming using CASAVA 1.8, matched libraries for a given sample were merged.

#### DNA sequencing:

Liver DNA from the eight animals was sequenced in paired-ends 2 × 101 bp on four lanes on an Illumina HiSeq 2000. Library preparation, DNA quantification, and sequencing were performed according to the manufacturers’ instructions using a TruSeq DNA Sample Prep Kit (Illumina, San Diego, CA), Agilent QPCR Library Quantification Kit (Agilent Technologies, Santa Clara, CA), TruSeq PE Cluster Kit v3 (Illumina, San Diego, CA) and cBot TruSeq SBS Kit v3 (Illumina, San Diego, CA). After quality checks and adapter trimming using CASAVA 1.8, matched libraries for a given sample were merged.

### Computational analyses

When not specified, analyses were performed with in-house Perl, Python and R scripts.

#### Genomic sequence analyses:

DNA sequences were aligned to the latest chicken genome assembly (Galgal4) using BWA v0.7.0 (Li and Durbin 2009) (*Command:* bwa aln). Sequences were then filtered based on mapping quality (*Command:* samtools view -bS -q 30). SAMtools v0.1.19 (H. Li *et al.* 2009a) *rmdup* (*Command:* samtools rmdup) was used to remove possible PCR and optical duplicates.

#### mRNA sequence analyses:

mRNA sequences were aligned with Tophat v2.0.5 (Kim *et al.* 2013) on the chicken reference genome Galgal4 as described in (Frésard *et al.* 2014) (*Command:* tophat–min-intron-length 3–max-intron-length 25000–max-deletion-length 1 -mate-inner-dist 200–read-realign-edit-dist 0–microexon-search). Uniquely mapped unduplicated sequences with a mapping quality greater than 30 were selected, using SAMtools v.0.1.19 (*Command:* samtools view -bS -q 30) and in-house Python script.

### Identification of mRNA editing candidates

Sequences were locally realigned and recalibrated before SNP detection, with GATK v1.6.11 for DNA (Van der Auwera *et al.* 2013) (*Commands:* GATK -T RealignerTargetCreator -R; GATK -T BaseRecalibrator -R -knownSites; GATK -T PrintReads -R -BQSR), and BamUtil (*Command:* bam recab) for RNA.

SAMtools v0.1.19 mpileup was used to detect SNPs between DNA and RNA samples from each individual (*Command:* samtools mpileup -d 10000). We set a maximum coverage of 10,000 reads in pileup for each calling to take into account as many reads as possible. SNPs were detected independently on each biological replicate. VCF files generated by SAMtools mpileup were then used for subsequent analysis. For each biological replicate, only variations where DNA was homozygous either for the reference allele or for the alternative allele (MAF = 1), and where RNA was heterozygous or homozygous for the alternative allele, were kept. Finally, we removed positions covered by less than 15 reads in both DNA and RNA alignments as well as triallelic sites.

### Impact of biases on mRNA editing detection

To explore whether each editing event was likely related to sequencing errors or alignment artifacts, we developed custom R and Perl scripts. We computed information related to: (1) Extremity bias, an editing event was considered as biased if the edited allele was mostly supported by the 10 first or last bases of reads in the RNA-seq read pileup [in accordance with previous studies (Kleinman and Majewski 2012; Frésard *et al.* 2015) we chose to consider only the distribution of the edited nucleotide position, to increase the stringency of the method]; (2) Strand bias, an editing event was considered as biased if the proportion of forward and reverse reads supporting it was markedly different (Δ >0.5); (3) Splice junction bias, an editing event was considered as biased if it was located within the region of a predicted splice site, *i.e.*, within 1–3 bases of the exon or 3–8 bases of the intron [to perform this analysis, we determined the annotation and localization of editing events in transcripts using Ensembl v71 Variant Effect Predictor (McLaren *et al.* 2010)]; (4) Homopolymer and low complexity bias, an editing event was considered as biased if the four neighboring positions harbored the same nucleotide or if it was falling in a single sequence repeat (SSR) [SSR were identified using SciRoKo (Kofler *et al.* 2007), the SSR patterns were investigated near candidate edited sites with an offset of ±3 bases]; (5) Multimapping bias, for each editing event, we generated a consensus 40 bp sequence centered on the edited allele, based on the pileup of RNA-seq reads harboring the edited alleles in a given sample. We then used fuzznuc (Olson 2002) to search for this sequence throughout the whole genomic DNA-seq reads including unaligned reads from the same sample. An editing event was therefore considered as biased if we found any match between its consensus surrounding sequence and genomic DNA-seq reads.

### Impact of biological replication on mRNA editing detection

For each tissue, we independently explored how reproducible each unbiased editing event was across the eight samples using custom R scripts. At each step, we computed the overall amount of events belonging to each class of substitution from DNA to RNA. Since our sequencing libraries were not strand-specific, the complement substitution of canonical editing events (*i.e.*, A-to-G for ADAR-mediated editing and C-to-T for APOBEC-mediated editing) were also considered as canonical (*i.e.*, C-to-T and G-to-A, respectively).

### Validation assays and editing yield quantification

#### DNA Sanger sequencing and RNA pyro-sequencing:

To assess whether the DNA genotype of candidate editing events was homozygous or not, we performed Sanger sequencing on the liver genomic DNA from the eight animals. We then assessed the mRNA genotype at these candidate sites on a PyroMark Q24 pyro-sequencer (Qiagen, Valencia, CA). Primers were designed with PyroMark Assay Design software (Supporting Information, Table S2). PCR products were prepared using the PyroMark PCR Kit (Qiagen, Valencia, CA) following the manufacturer’s instructions. Data were analyzed with PyroMark Q24 v1.0.10 using default parameters.

#### Experimental designs used to test the effect of age, sex, genetic background, and feeding on mRNA editing level:

To measure the impact of age, sex, genotype, and feeding on mRNA editing level, we used different independent experimental designs with animals contrasted for these factors: (1) Genotype, broilers (N = 8) and layers (N = 8) in two unrelated experimental designs (*i.e.*, N = 2 × 16 in all); (2) Age, prepuberal (N = 8) and postpuberal (N = 8) layers; (3) Feeding, broilers slaughtered after 24 hr fasting (N = 8) or broilers fed *ad libitum* (N = 8) in two independent studies (*i.e.*, N = 2 × 16 in all); (4) Sex, female (N = 8) and male (N = 8) layers. Liver DNA and liver RNA and/or adipose RNA were extracted and quality checked as described before (see *Tissue collection and library preparation*). We then performed both liver genomic DNA and liver and/or adipose mRNA pyro-sequencing following the aforementioned procedure for five edited sites (*i.e.*, two liver-specific, one adipose-specific, two common to both tissues) previously validated in our main experimental design. The pyro-sequencing signal at the edited position in mRNA was standardized according to the signal obtained on DNA to avoid amplification and sequencing biases. The editing level was computed as the ratio between signals for the mRNA edited allele and allele on genomic DNA. We finally tested the effect of each factor on RNA editing level at the five selected sites using a two-sided unpaired homoscedastic Student *t*-test. Statistical analyses were performed on R 3.2.0 using t.test (two sided unpaired homoscedastic Student *t*-test) functions from the stats package and graphical visualizations plotted using the ggplot2 package.

### In silico prediction of RNA editing impact on protein structure and function

To predict the putative effect of RNA editing on protein structure and function, we first identified genomic structures likely to be impacted using Ensembl v71 Variant Effect Predictor (McLaren *et al.* 2010). Focusing on missense coding editing events, we then recovered orthologous protein sequences from *Gallus gallus*, *Bos taurus*, *Rattus norvegicus*, *Mus musculus* and *Homo sapiens* to carry out multi-alignment. We finally used the SIFT prediction tool (Kumar *et al.* 2009), which is based on both sequence homology and the physical properties of amino acids, to quantify the potential impact of coding editing events on protein structure and function.

### Data availability

Liver and adipose mRNA-seq raw data are available on Sequence Read Archive under accession SRP042257. Liver genomic DNA-seq raw data are available on Sequence Read Archive under accession SRP042641.

## Results

### High-throughput sequence analyses

Liver DNA and liver and white adipose tissue RNA were extracted from eight 16-week-old male chickens, and paired-end sequenced on an Illumina HiSeq2000. After alignment on the current genome assembly Galgal4, and filtering on mapping quality and multimapping, we conserved an average of 157 million DNA-seq reads, 30 million liver mRNA-seq reads and 38 million adipose RNA-seq reads per sample. On average, 93.5% of the genome was covered by at least 15 DNA-seq reads, and 14.3% and 18.4% by at least 15 mRNA-seq reads from liver and white adipose tissue (WAT), respectively.

### mRNA editing detection and initial filtering

**Figure 2:**
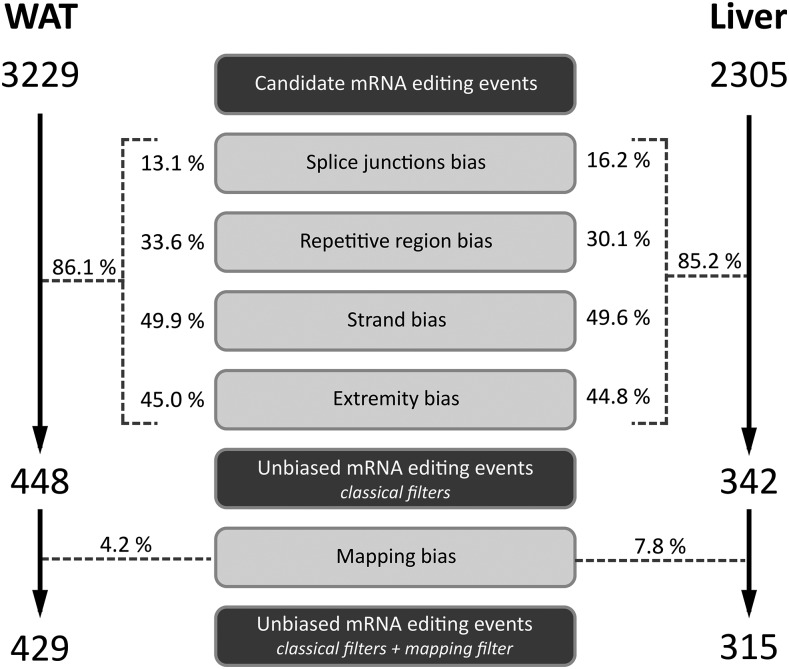
Impact of sequencing and mapping biases on mRNA editing discovery. Contribution of random or systematic sequencing biases and mapping artifacts to the false discovery of mRNA editing events using combined mRNA and DNA sequencings are given as a fraction (%) of the intial pool of candidate editing events subject to each source of bias in each tissue. WAT, white adipose tissue.

For each biological replicate, a base modification A (DNA base) → B (RNA base) was considered as a candidate mRNA editing event if: (1) the genotype inferred for the genomic DNA was homozygous AA with a minor allele frequency equal to 1 (*i.e.*, all the reads were supporting a unique allele on the genomic DNA sequence); (2) the genotype inferred for the mRNA sequence was biallelic heterozygous AB or homozygous BB; (3) the position was covered by at least 15 reads of both genomic DNA and mRNA sequences; and (4) the mRNA editing event did not imply an insertion or deletion event. A total of 3229 and 2305 positions met these criteria in WAT and liver, respectively (Figure 2).

### Impact of sequencing and mapping artifacts on false editing discovery rate

In order to increase accuracy in the detection of editing events and to reduce the amount of false positives, a standard procedure consists to apply different *ad hoc* filters to remove suspicious candidates presenting error-prone splice junction bias, strand bias, extremity bias, splice bias, or repetitive region bias. We conducted a first analysis aimed at assessing the amount of candidate mRNA editing events that were spurious with respect to each of these biases. This revealed that splice junction bias concerned about 15% of candidates in both WAT and liver (Figure 2). While the amount of repetitive region biased mRNA editing sites exceeded 30% of the candidate positions, this value increased to more than 45% considering strand or extremity biased positions (Figure 2) in both tissues. Considering these sequencing and mapping artifacts together, we showed that more than 85% candidates were subject to at least one source of bias. Thus, using these classical filters (*i.e.*, usually applied in editing screening studies), the amount of candidate mRNA editing events dropped from 3229 and 2305 to 448 and 342 in WAT and liver respectively.

### Correction for multimapping based on DNA-seq raw sequences

Even if somehow taken into account during or after mRNA-seq read mapping, the reads multi-mapping may still have a great impact on mRNA editing false discovery rate because of gaps and miss-assemblies in the reference genome. To carefully control this artifact, we used the approach we first introduced in our previous study (Frésard *et al.* 2015), consisting of aligning back mRNA-seq reads harboring a candidate mRNA editing site on corresponding individual DNA-seq reads, independently if they were aligned or if not onto the genome. We revealed that among the 448 and 342 remaining candidates, 4.2% and 7.8% were multimapping-related false positives in WAT and liver, respectively (Figure 2). Finally, considering both previously described filters and this last filter dealing with multimapping, we ended up with 429 and 315 unbiased mRNA editing sites in WAT and liver, respectively.

### Impact of biological replication on canonical mRNA editing event identification

As previously mentioned in the introduction, the number of biological replicates (N) taken into account in mRNA editing screening studies based on RNA-seq data is highly variable, with N ranging from 1–6. As depicted in Figure 1, when the number of biological replicates is lower than 2, the total number of editing events is extremely variable, ranging from 15 (Picardi *et al.* 2012) to 1,586,270 (Bazak *et al.* 2014). This number decreases drastically between 40 (Frésard *et al.* 2015) and 253 (Gu *et al.* 2012) when N ≥3, suggesting that considering biological replicates partly counteracts the lack of filters dealing with sequencing and mapping artifacts. Nevertheless, substantial variability remains between studies, likely related to differences in the artifacts considered and the stringency of bioinformatics filters, and to the biological context (*e.g.*, tissue, species) of each study.

**Figure 3:**
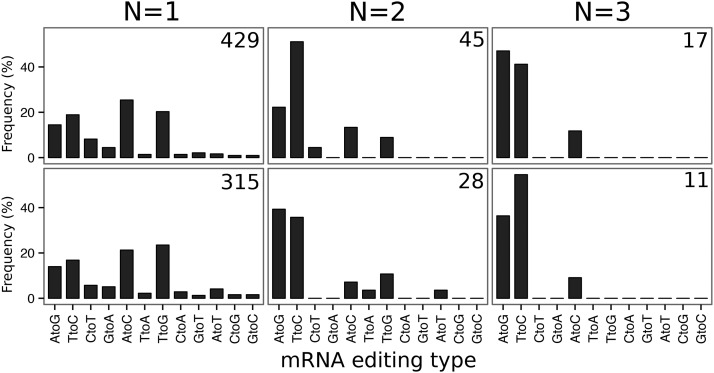
Impact of biological replication on mRNA editing discovery. Distribution (in %) of unbiased mRNA editing events across the 12 classes of substitution according to the number of replicates they are detected in, ranging from N = 1 to N = 3, in white adipose tissue (WAT) and liver. The first two classes (AtoG and TtoC) are associated to ADAR-mediated RNA editing, and the next two (CtoT and GtoA) to APOBEC-meditated RNA editing. At the top-right of each graph, the total number of RNA editing events detected for a given number of replicates is shown. ADAR: Adenosine deaminases acting on RNA. APOBEC: Apolipoprotein B mRNA editing enzyme, catalytic polypetide-like.

**Figure 4:**
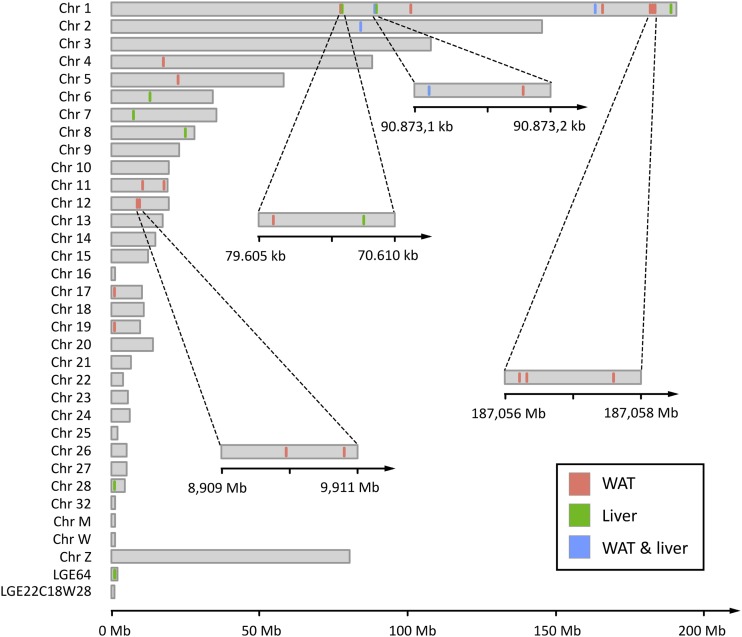
Position of mRNA editing events across the chicken genome. WAT: White adipose tissue.

With this observation in mind, we characterized the 429 and 315 mRNA editing events previously detected according to the number of biological replicates they were detected in, and the class of base substitutions they belong to. We differentiated canonical mRNA editing events (A-to-I and C-to-U interpreted as A-to-G and C-to-T by genome analyzers, and corresponding to editing events catalyzed by ADARs and APOBECs) from noncanonical events that are not explained by any of the two known editing mechanisms. As the RNA-seq libraries used in this study were not strand-specific, we also considered complement bases of canonical changes as canonical editing events (*i.e.*, T-to-C and G-to-A). As can be seen in Figure 3, in the sets of 429 and 315 unbiased mRNA editing events detected in at least one individual described above, the amount of transversions (*i.e.*, pyrimidine-to-purine, and purine-to-pyrimidine, 54.1% in WAT and 58.0% in liver) was greater than the amount of transitions (*i.e.*, pyrimidine-to-pyrimidine, and purine-to-purine). Adding restriction based on the number of biological replicates these events must be detected in, the amount of transversions progressively decreased from more than 50% considering no replication (N = 1), to 25% (N = 2) and finally to 10% (N = 3) requiring editing events to be detected in at least three replicates. Considering that mRNA editing events were reproducible across at least three biological replicates, we finally conserved 19 and 11 positions in WAT and liver respectively, comprising one noncanonical transversion event in each tissue (Table 2), distributed across 13 chromosomes (Figure 4). Among these 27 unique events, three were common to both tissues. While most of these events were spatially isolated from each other, some of them were clustered in short genomic regions spanning a few bp (Figure 4), especially on chromosome 1 (three mRNA editing events in a window of 1.391 bp downstream *NOX4* in WAT, and two in a window of 26 bp downstream *MPZL1* and *BRP44* in liver) and chromosome 12 (2 mRNA editing events in a window of 951 bp in *FLNB*).

**Table 2:** mRNA editing screening in adult chicken liver and adipose tissue

	Chromosome	Position	DNA Allele	RNA Allele	Canonical	Validation in Sanger	Validation in PyroMark	Replicates*a*	Gene Names*b*	Localization*c*
WAT	1	79605543	T	C	yes			3	PLA1A, POPDC2	Downstream gene
	1	*90873162*	T	C	yes			3	MPZL1, BRP44	Downstream gene
	1	103511385	T	C	yes			6	GRIK1	Intron
	**1**	***167109833***	A	G	yes	yes	yes	3	COG3	Exon (missense)
	1	169769193	A	G	yes			3	THSD1	Downstream gene
	1	187056174	A	G	yes			3	NOX4	Downstream gene
	1	187056183	A	G	yes			3	NOX4	Downstream gene
	1	187057565	A	G	yes			3	—	Intergenic
	**2**	***86000926***	T	C	yes	yes	yes	5	NDUFS6	Upstream
	4	17996546	T	C	yes			4	—	Intergenic
	5	22958596	T	C	yes			3	DGKZ	Intron
	11	10634278	A	G	yes			5	CES1	Exon (missense)
	11	19169664	A	C	no	no	no	3	DHODH, IST1	Upstream, Intron
	**12**	**8909946**	A	G	yes	yes	yes	6	FLNB	Exon (missense)
	12	8910897	A	G	yes			5	FLNB	Intron
	17	4705	T	C	yes			3	—	Intron
	17	94999	C	T	yes			6	—	Intergenic
	18	28378	T	C	yes			6	ZNF302	Upstream
	19	4812610	C	T	yes			4	CCLI8	Intron
Liver	**1**	**79608260**	T	C	yes	yes	yes	7	POPDC2, PLA1A	Downstream gene
	1	*90873162*	T	C	yes			3	MPZL1, BRP44	Downstream gene
	1	90873188	T	C	yes	yes	yes	4	MPZL1, BRP44	Downstream gene
	**1**	***167109833***	A	G	yes	yes	yes	5	COG3	Exon (missense)
	1	193343226	A	G	yes			6	MADPRT, ART7B	Upstream
	**2**	***86000926***	T	C	yes	yes	yes	5	NDUFS6	Upstream
	6	13236424	A	G	yes			3	KCNMA1	Exon (missense)
	**7**	**7564791**	A	G	yes	yes	yes	5	MYO1B	Intron
	8	25588113	T	C	yes			3	—	Exon (synonymous)
	28	518606	A	C	no	yes	no	3	HNRNPM	Missense
	LGE64	615592	T	C	yes			4	—	Intron

In italics, mRNA editing events common to both tissues; in bold, mRNA editing candidate events subjected to Sanger genomic DNA sequencing and cDNA pyrosequencing; Underlined, mRNA editing events annotated as “coding - missense” and located in four different genes.

a Number of samples in which the mRNA editing event is detected.

b Name of the gene impacted by the mRNA editing event or name of the closest genes (<10 kb) if the mRNA editing event is falling in an intergenic region.

c Localization of the mRNA editing event inside genomic features, as predicted by Variant Effect Predictor (McLaren *et al.* 2010). If the mRNA editing event is falling inside a coding region, its impact on gene product is given in brackets.

### Validation of candidate mRNA editing events

To assess the validity of some of the edited positions detected using the high-throughput screening approach, we performed Sanger sequencing on DNA to confirm their homozygous genotype and pyrosequencing on RNA to validate the mRNA base recoding at the corresponding position on the transcriptome. The validation revealed that noncanonical mRNA editing events were false positives either related to genomic SNPs undetected using genomic DNA-seq data for the one in WAT, or to unbalanced allelic expression not detected through mRNA-seq data for the one in liver (Table 2). Following the same approach, we selected five canonical events for validation: two out of the three candidates detected in both tissues, two specific to WAT, and one liver-specific. We first confirmed their homozygous status on genomic DNA and the mRNA recoding at these positions was then confirmed by mRNA pyrosequencing (Table 2) using samples from the tissue they were detected in.

### Functional characterization of liver and white adipose editomes

**Figure 5:**
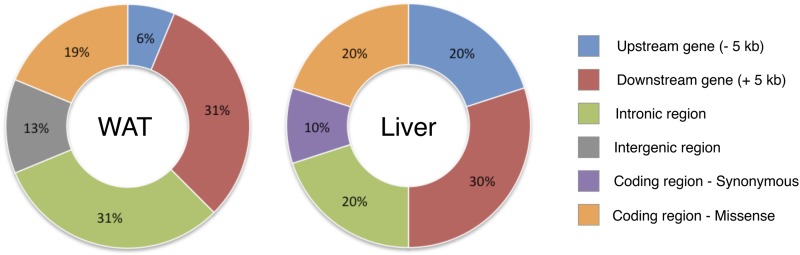
Distribution of mRNA editing events across genomic features. Annotations were assessed using Ensembl v71 Variant Effect Predictor (McLaren *et al.* 2010).

**Figure 6:**
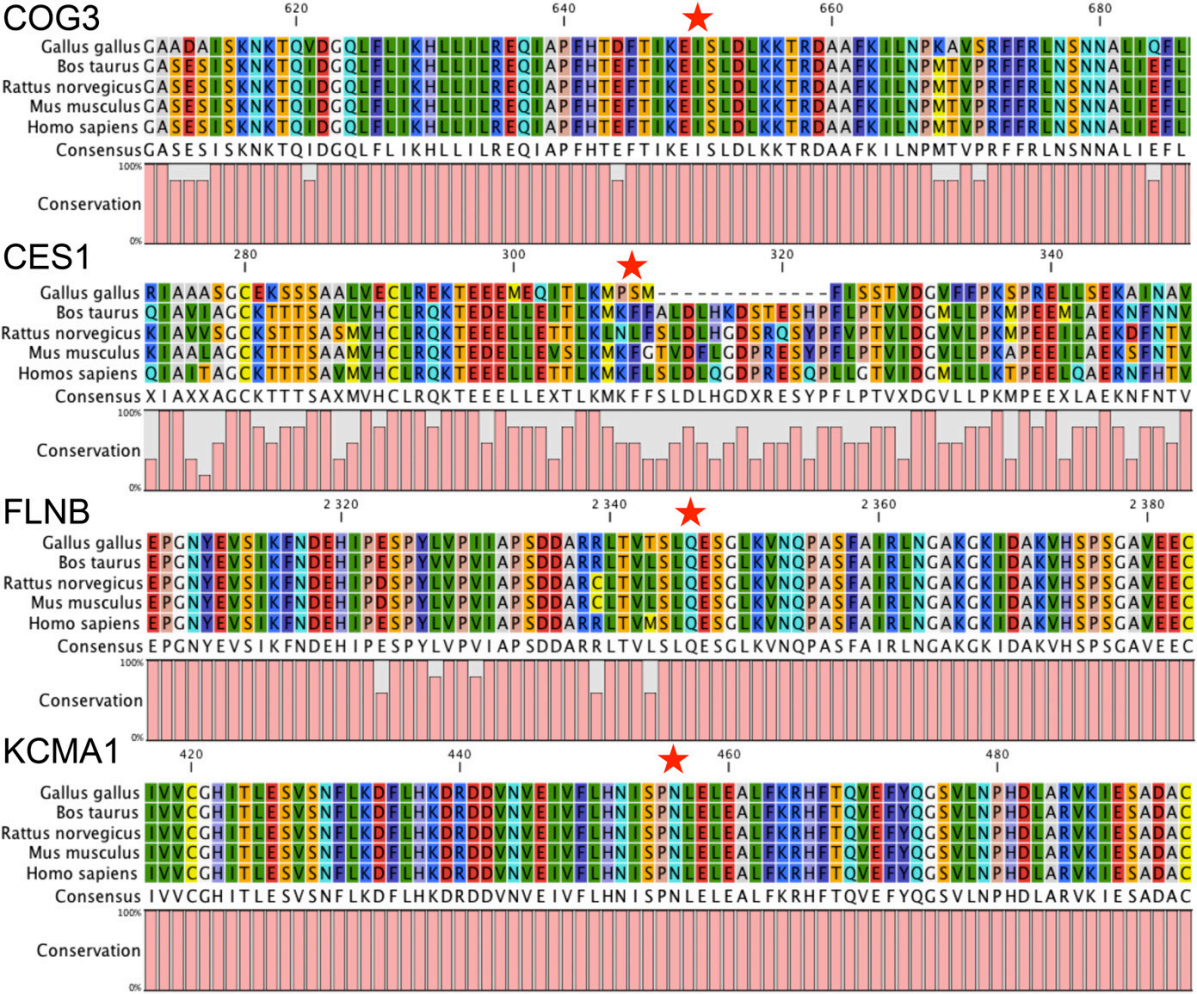
Multispecies protein sequence alignments for coding mRNA editing events. The red stars indicate the position of the amino acid impacted by coding mRNA events. The overall conservation across sequences is depicted below each alignment. The mRNA editing event impacting *COG3* was detected in both white adipose tissue (WAT) and liver, while the ones impacting *CES1* and *FLNB* were WAT-specific, and the one impacting *KCMA1* was specific to liver.

Functional annotation of the 25 unique canonical mRNA editing events using Variant Effect Predictor revealed that most of them were located in noncoding regions, since 81% and 70% were situated in either 5 kb gene flanking regions, intronic regions or intergenic regions in WAT and liver, respectively (Figure 5). Only five unique mRNA editing events were annotated as coding. Among these, four were likely to impact the mature protein: one common to both tissues on *COG3*, two WAT-specific on *CES1* and *FLNB*, and one liver-specific on *KCMA1* (Table 2). Focusing on these four missense mRNA editing events, we conducted a fine functional annotation analysis using the SIFT software to assess the impact of the amino acid substitution on these proteins. This revealed that none of these mRNA editing events was likely to be deleterious. Nevertheless, after carrying out multiple species protein alignments considering *G. gallus*, *B. taurus*, *R. norvegicus*, *M. musculus* and *H. sapiens*, we showed that except for *CES1*, these missense mRNA editing events were impacting highly conserved amino acid residues (Figure 6).

### Impact of genetic background, age, sex, feeding, and tissular context on editing level

**Figure 7:**
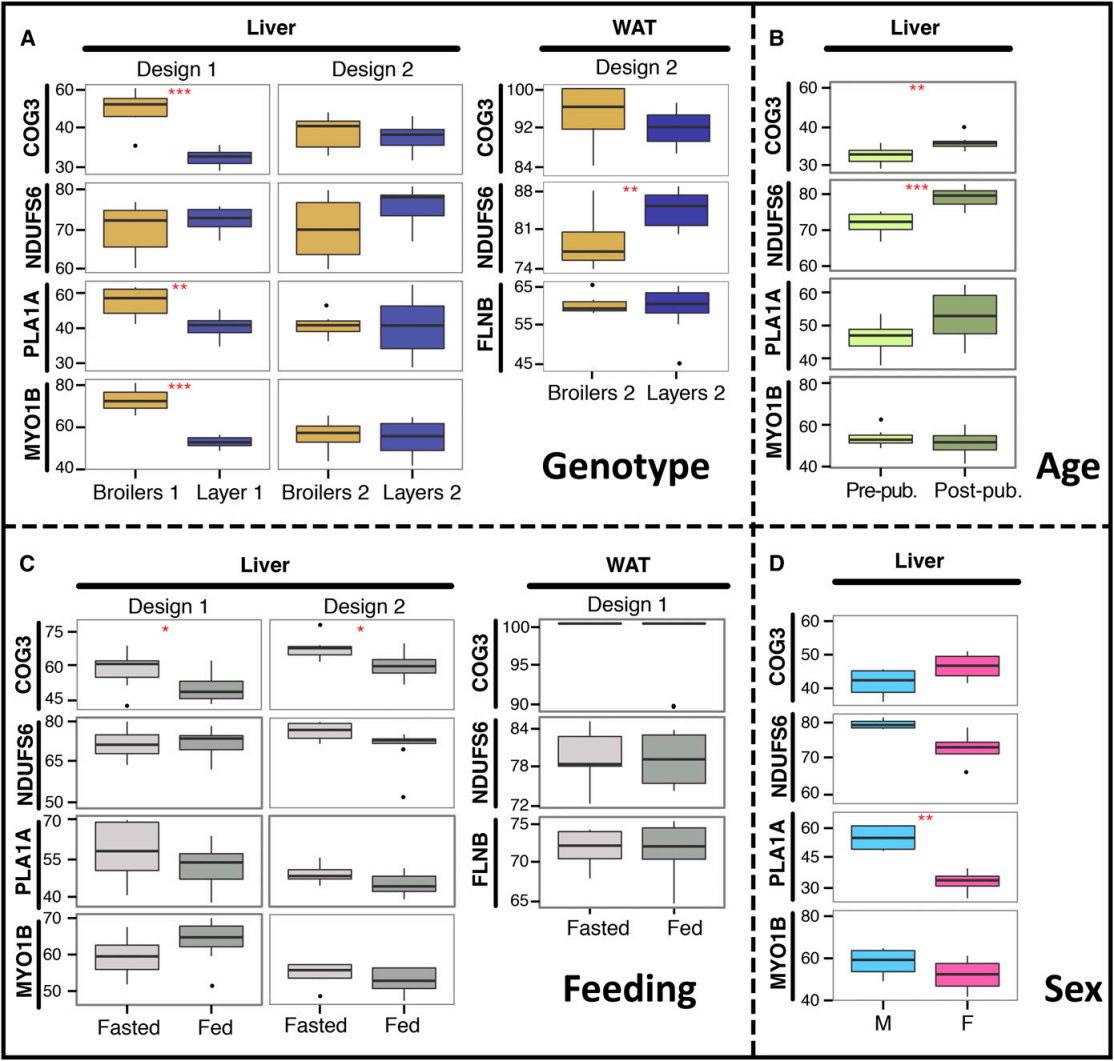
Impact of genetic background, age, feeding conditions, and sex on mRNA editing level. Editing level (in %) at five genomic positions, in white adipose tissue (WAT) and liver according to (A) genetic background, (B) age, (C) feeding conditions, and (D) sex. Each boxplot shows the distribution of editing levels (% of the edited allele) across N = 8 biological replicates. * *P* <0.05, ** *P* <0.01, *** *P* <0.001, unpaired two-tailed Student *t*-test. F, Females; M, Males; Post-pub, postpuberal animals; Pre-pub, prepuberal animals.

To test the impact of genetic background, age, feeding conditions and sex on the mRNA editing level, we considered the aforementioned subset of five validated canonical mRNA editing events. We tested the effect of: (1) the genotype, comparing mRNA editing level in liver and WAT between broilers and layers in two independent experimental designs (Figure 7A); (2) the age, comparing mRNA editing level in liver between prepuberal and postpuberal chickens (Figure 7B); (3) the feeding conditions, comparing mRNA editing level in both tissues between chickens slaughtered after 24 hr fasting and feeding *ad libitum*, in two independent experimental designs (Figure 7C); (4) the sex, comparing mRNA editing level in liver between roosters and hens (Figure 7D). To test each effect, we performed both genomic DNA Sanger sequencing and mRNA-derived cDNA pyro-sequencing on eight independent biological replicates in each group. Our analysis on liver revealed a significant effect of genotype on mRNA editing level in the first design for three different positions among the four tested (*P*-values: 1.26 × 10^−5^, 1.52 × 10^−3^ and 1.33 × 10^−6^, Figure 7A and Table S1). In WAT, we highlighted one out of the three tested positions for which the editing level was significantly different between broilers and layers (*P*-value: 9.44 × 10^−3^, Figure 7A and Table S1). Interestingly, while the general tendency was a greater editing level in broilers in comparison to layers in the liver (*COG3*, *PLA1A* and *MYO1B*), this trend was reversed for the highlighted site in WAT (*NDUFS6*). Regarding the age, we observed a significant effect on editing levels for two sites in liver (Figure 7B and Table S1). Concerning the effect of sex, one mRNA editing event showed a significant increase of mRNA editing level between males and females in liver (*P*-value: 1.90 × 10^−3^, Figure 7D and Table S1). Finally, by analyzing the effect of the feeding conditions, we highlighted a significant increase of the mRNA editing level after a 24 hr fast for one site in liver in two independent experimental designs (Figure 7C and Table S1). Noticeably, for the edited position located on *COG3*, the mRNA recoding level was significantly impacted by genetic background, age, and feeding.

## Discussion

To achieve whole-transcriptome screening for mRNA editing events in chicken liver and adipose tissue, we detected discrepancies between genomic DNA and mRNA sequences using matched genomic DNA-seq and mRNA-seq data in several biological replicates. Since 2009 (J. B. Li *et al.* 2009b), similar approaches based on mRNA-seq have been extensively used to characterize mouse, human, and chimpanzee editomes in different tissues. According to the literature, the extent of mRNA editing is highly variable with estimates ranging from dozens to millions, even when comparing studies focusing on the same tissue in the same species. After in-depth reading of mRNA editing screening studies, we highlighted that, despite recommendations for the use of rigorous bioinformatics pipelines to characterize editomes (Kleinman and Majewski 2012; Lin *et al.* 2012; Pickrell *et al.* 2012), many recent studies have neglected the most reviewed sequencing and mapping artifacts related to mRNA-seq, such as strand bias, read extremity bias, splice junction bias, and low complexity region bias (Table 1). In our study, these random or systematic biases each impacted between 13% and 50% of the initial set of differences between the DNA and RNA we detected. Overall, we showed that almost 90% of the candidate mRNA editing events initially detected were likely to be false positives arising from one of these artifacts, revealing how huge their impact is on the false discovery rate, as previously reported (Pickrell *et al.* 2012; Lagarrigue *et al.* 2013). Interestingly, the false positive rate we report is in agreement with the observations of Pickrell and collaborators (Pickrell *et al.* 2012), suggesting that among the 28,766 editing events detected in the study of Li and collaborators (Li *et al.* 2011), roughly 90% were likely false positives emerging from sequencing errors and mapping artifacts.

Even if multimapping is considered during mRNA-seq read mapping, this artifact could still be of a great impact on mRNA editing false discovery rate. Indeed, current genome assemblies used to map short reads from high-throughput sequencing experiments, even of high quality for human, mouse or chicken, are still presenting missing sequences as well as misassembled regions (Groenen *et al.* 2011, Genovese *et al.* 2013a,b). Since these regions might harbor sequences that are paralogous to properly assembled parts of genomes, this ultimately leads to shallow identification of multimapped short reads when only the reference sequence is considered. To by-pass this error-prone issue for the identification of mRNA edited sites, in this study we used an approach first introduced in our previous study (Frésard *et al.* 2015), consisting of looking for mRNA sequences spanning edited sites in raw genomic DNA sequences, and confirmed its efficiency. In our study, up to 8% of the initial candidate differences between RNA and DNA were false positives related to errors and assembly issues in the chicken genome. This result suggests that thousands of edited sites reported in primates and mice mRNA editing screening studies could be partly attributed to false positives resulting from spurious handling of multi-mapped mRNA-seq short reads. More strikingly, a huge proportion of mRNA editing screening studies are solely based on mRNA-seq data, and do not consider individual matched genomic DNA-seq data for the samples analyzed. In these studies, candidate edited sites are filtered using the positions of known SNPs referenced in databases such as dbSNP, rather than considering individual polymorphisms, thereby fully occulting inevitable individual specific genomic variations. Even in the case of clonal mice strains, considering a consensus strain-specific genomic sequence as exposed by Danecek and collaborators (Danecek *et al.* 2012) indubitably leads to edited event false calls that arise from somatic mutations, as we previously showed in our study on mice, invalidating 25% of mRNA editing candidates arising because of unreferenced genomic SNP (Lagarrigue *et al.* 2013).

To further limit the amount of false positives among mRNA edited sites and to focus on biologically meaningful mRNA editing events, an obvious approach consists of considering biological replication. Surprisingly, a lot of mRNA editing screening studies report events without considering reproducibility across samples (Figure 1 and Table 1). Although it is likely that mRNA editing is partly an individual-specific phenomenon (Gommans *et al.* 2009), short read sequencing technologies are error-prone when it comes to focus on slight variations and are not mature enough to allow the investigation of private editing events. Therefore, biological reproducibility is uncontestably required in this scope. In our study, even after filtering to properly account for systematic and random sequencing artifacts as well as multimapping, we were still detecting more than 50% of noncanonical mRNA editing events. Hitherto, the attempts by other groups to validate such types of mRNA recoding using targeted Sanger sequencing were unsuccessful (Piskol *et al.* 2013), clearly ascertaining that they arise from unconsidered artifacts. When focusing only on editing events detected in at least three biological replicates, the proportion of canonical events increased to 90% in the present work. If noncanonical recoding events were not related to artifacts, we would not have expected such an enrichment, which further confirms that they are false positives. With respect to this hypothesis, we invalidated the two noncanonical events that we were still detecting after considering biological replication. Overall, our results suggest that filtering to consider false positives arising from mapping artifacts and sequencing errors, even if mandatory, is not sufficient to remove all spurious editing events. While focusing on the most biologically meaningful recoding events that are shared between individuals, considering biological replications is decisive regarding the amount of false positives in RNA editing screening studies based on current high-throughput sequencing technologies.

Altogether, considering filters dealing with systematic and random sequencing errors, multimapping, mapping artifacts, and biological replication, the number of edited sites dramatically felt from 3.229 and 2.305 candidates to 19 and 11 robust events in WAT and liver, respectively. Even using highly stringent filters, two noncanonical false positives were still detected, once again suggesting that hard filtering and biological replication are still mandatory when working with current short-sequencing technologies. Even if a greater sequencing depth would have allowed the detection of a slightly higher number of edited sites, our work reveals that the extent of mRNA editing is, at least in chickens, far below what has been previously shown in most screening studies on humans, mice and chimpanzees. Interestingly, most of the studies reporting an amount of mRNA editing events close to that which we have recorded have been conducted on healthy tissues rather than immortalized cell lines or tumors, and considered only sites edited in at least two biological replicates (Gu *et al.* 2012; Lagarrigue *et al.* 2013; Frésard *et al.* 2015). The huge variation in the extent of mRNA editing between our study and other screening studies in the literature could be explained in different ways. First of all, it is likely related to the differences in the stringency of filters applied and the false positive rate. Second, most of the mRNA editing screening studies were carried out using transformed cell lines or cancer tissues (Table 1), and the extensive mRNA editing reported may reflect real biological changes. Indeed, ADARs and APOBECs may become more active during tumorigenesis, and may consequently increase mRNA editing, as it has been highlighted in some cancer cell lines (Galeano *et al.* 2012). Third, it could also be explained by the huge structural differences between mammalian and sauropsidian genomes. ADAR-mediated A-to-I mRNA editing occurs in regions of double stranded RNA (dsRNA), yet approximately half of a typical mammalian genome contains highly repetitive sequences (de Koning *et al.* 2011) such as retrotransposons, short interspersed nuclear elements (SINEs), and long interspersed nuclear elements (LINEs). While these sequences are often repeated in reverse tandems, they may generate dsRNA structures that could be subsequently edited by ADARs (Nishikura 2010). In chickens, since the amount of repetitive sequences across the genome falls below 15% (Wicker *et al.* 2005; Schmid *et al.* 2015), it is expected that less A-to-I editing events occur.

At the end, among the 25 unique canonical mRNA editing events we report, only 3 (*i.e.* 13%) are common to both tissues : 1 located downstream *MPZL1* and *BRP44*, 1 located in *COG3* and 1 located upstream *NDUSF6*. Comparisons with our previous study highlighted that only four mRNA edited sites detected in chicken whole embryos were also found in mature WAT or liver. Surprisingly, only the edited sites located in *COG3* and upstream *NDUFS6* were common to WAT, liver and whole embryos. These results are comparable to those reported by Danecek *et al.* and Lagarrigue *et al.*, revealing a significant amount of tissue-specific edited sites (Danecek *et al.* 2012; Lagarrigue *et al.* 2013). Interestingly, while no homolog of *APOBEC1* has been characterized in the chicken genome (Conticello *et al.* 2005), all of the APOBEC-mediated C-to-U mRNA editing candidate sites that we initially detected were discarded along the filtering pipeline, confirming that this specific mRNA editing mechanism is missing in chickens. We also found that some of the mRNA editing events we detected were localized on mRNA editing clusters spanning regions of a few kb. This observation is supported by our current knowledge regarding the mechanistic basis of ADAR-mediated A-to-I mRNA editing, which occurs unspecifically in dsRNA, and doesn’t involve a specific mooring sequence, as is the case for APOBEC-mediated C-to-U mRNA editing (Nishikura 2010).

Overall, most of the mRNA editing events we detected fall in noncoding regions (*i.e.*, 10 kb upstream or downstream of genes, in introns, or in intergenic regions). Since these regions are expressed, they are either corresponding to poorly annotated genomic regions, nonmature mRNAs, or unannotated noncoding RNAs in which RNA editing is known to occur (Picardi *et al.* 2014). Out of these 25 unique mRNA editing events, five are located in coding sequences and only four are nonsynonymous, impacting the sequence of *COG3*, *CES1*, *FLNB*, and *KCNMA1*. Interestingly, the edited sites falling in *COG3* and *FLNB* were already described in mammalian species (Levanon *et al.* 2005; Shah *et al.* 2009; Danecek *et al.* 2012; Holmes *et al.* 2013; Stulic and Jantsch 2013), revealing that some edited positions are conserved throughout evolution between birds and mammals. Except for *CES1*, our analyses show that each of these coding mRNA editing events impact upon highly conserved regions in the protein sequence, as well as highly conserved amino acid residues.

Our analysis finally shows that the mRNA editing level is impacted by various genetic and environmental factors such as genetic background, age, feeding conditions, and sex. While the genetic background and age influence the editing level at almost all of the mRNA editing sites tested, feeding conditions and gender tend to affect fewer positions. The impact of aging on mRNA editing has already been reported in a few studies on mammals (Wahlstedt *et al.* 2009; Shtrichman *et al.* 2012; Venø *et al.* 2012), as well as in our previous study on chicken embryos (Frésard *et al.* 2015). In agreement with most of these studies, we confirm that the level of edited transcripts increases with age, whatever the recoded site considered. Nevertheless, an in-depth unbiased whole-transcriptome exploration of the basis of the spatio-temporal regulation of mRNA editing in vertebrates is still needed. We also observed a significant effect of genotype on the liver mRNA editing level indicating another level of regulation. Indeed, in one of the designs used to assess the effect of genetic background, three out of four edited isoforms (in *COG3*, *PLA1A* and *MYO1B*) were 1.5–2-fold more frequent in broilers’ livers compared to layers’ livers. *PLA1A* encodes for the phosphatidylserine-specific phospholipase A1, which is mostly synthesized in the liver and is implicated in the release of free fatty acids and lysophosphatidic acid, which acts as a lipid mediator in cell signaling. While it is established that this lipase does not catabolize triglycerides, its role in global cellular processes is still poorly understood (Aoki *et al.* 2002). *MYO1B* encodes for the widely expressed myosin 1B motors that function in endocytosis, membrane trafficking, membrane retraction, and mechano-signal transduction. Even though the physiological landscape of myosin 1B is not yet fully understood, some authors have hypothesized its potential role on myogenesis (Wells *et al.* 1997; Redowicz 2007). While muscle mass stands as one of the most divergent phenotypic traits between layers and broilers, mRNA editing in *MYO1B* could be part of the transcriptional basis leading to differences in muscle development between these strains, but it remains to be seen whether this site is also differentially edited in muscular tissues between broilers and layers. Finally, *COG3* has a general cellular function related to the structure and function of the Golgi, as further described below. Since the editing levels at the sites located in *COG3*, *PLA1A* and *MYO1B* are differential between broilers’ and layers’ livers, and because liver is a multi-function organ involved in many physiological processes, we hypothesized that they might be implicated in cellular and developmental processes leading to physiological differences between these two chicken strains. The genetic regulation of mRNA editing level at these sites could be linked to mechanisms acting in *trans*, involving ADARs, and further analyses comparing ADAR expression and activity between these two chicken genetic backgrounds are mandatory to investigate this hypothesis. They could also be regulated by *cis*-acting mutations impacting surrounding mRNA sequences and secondary structures, as has been recently suggested regarding mRNA recoding in *Drosophila* (Sapiro *et al.* 2015).

While the extent of mRNA editing appears limited at the transcriptome scale in chicken liver and WAT, our results suggest that this phenomenon could be tightly regulated. Indeed, the I/V nonsynonymous recoding event impacting *COG3* is not only conserved in mammals but is also under the influence of the genetic background, age, and feeding conditions. For this last factor, this edited site was the only one impacted, in two independent designs, which suggests that this observation is highly reliable. It is also noticeable that *COG3* mRNA is almost exclusively edited in WAT, with in average 95% (in the “genotype” design) or 100% (in the “feeding” design) of the isoform edited, in contrast with observations in the liver transcriptome. This further suggests that these different isoforms are likely harboring different physiological functions and that ADAR-mediated mRNA editing could act in a highly tissue-specific manner, as previously shown (Song *et al.* 2004), in a way that is similar to APOBEC1-mediated *APOB* mRNA editing, which ultimately leads to the synthesis of two APOB isoforms – APOB100 in the liver, and APOB48 in the small intestine – with distinct physiological functions. Since the edited site in *COG3* has been conserved throughout the evolution of vertebrates, and it is tightly regulated by multiple genetic and environmental factors, it is likely to have a functional role on the encoded protein. *COG3* is one of the eight proteins of the oligomeric Golgi (*COG*) complex. The *COG* complex is involved in intra-Golgi retrograde trafficking and in membrane trafficking in eukaryotic cells (Loh and Hong 2004; Zolov and Lupashin 2005). Mutations affecting *COG* subunits disturb both the structure and function of the Golgi (Ungar *et al.* 2002), and have been reported in congenital disorders of glycosylation (Kodera *et al.* 2015). These different studies show an important role of the *COG* complex in eukaryotic cells. It is known to be an evolutionarily conserved multi-subunit protein complex, but its exact cellular function remains elusive. While this edited site in *COG3* is conserved across stages (embryo and adult stages in chicken) and species (human, mouse, rat and chicken), additional work is required to decipher its potential role in *COG3*’s functions, and potentially on membrane trafficking pathways.

This study, which is complementary to our previous study conducted on chicken embryos, is the first describing the mRNA editing landscape in adult chickens. From a methodological point of view, we show how huge the impact of sequencing biases and mapping artifacts can be on the discovery of mRNA editing events if not properly considered. Moreover, we show the importance of considering biological replication with high-throughput sequencing data to filter spurious candidates, allowing focusing on the most biologically meaningful mRNA editing events. From a biological point of view, even if we cannot claim that we are exhaustive, our results support the evidence that the extent of mRNA editing is limited in chickens and restricted to ADAR-mediated events. We also ascertain that some editing sites are conserved throughout the evolution of vertebrates. Our study finally shows that mRNA editing levels are strongly affected by genetic background and age and, to a minor extent, by feeding conditions and sex, which provides new insights into our comprehension of mRNA editing functions in vertebrates in relation to genetics and environmental components.

## Supplementary Material

Supporting Information
