# Supplementary material for: The Extent of mRNA Editing Is Limited in Chicken Liver and Adipose, but Impacted by Tissular Context, Genotype, Age, and Feeding as Exemplified with a Conserved Edited Site in COG3
Source: G3 (Bethesda). 2015 Dec 2;6(2):321–35. doi: 10.1534/g3.115.022251 (PMC4751552; doi:10.1534/g3.115.022251)
Supplement: Supporting Information [file supp_6_2_321__index.html]

The Extent of mRNA Editing Is Limited in Chicken Liver and Adipose, but Impacted by Tissular Context, Genotype, Age, and Feeding as Exemplified with a Conserved Edited Site in COG3 — Supporting Information 

# The Extent of mRNA Editing Is Limited in Chicken Liver and Adipose, but Impacted by Tissular Context, Genotype, Age, and Feeding as Exemplified with a Conserved Edited Site in COG3

## Supporting Information for Roux *et al.*, 2016

**Files in this Data Supplement:**

- Table S1 - Detailed results of statistical analyses revealing the effect of genetic and environmental factors on editing level. (.xlsx, 46 KB)
- Table S2 - Description of primers used to validate candidate edited sites and to assess the effect of genetic and environmental factors on editing level. (.xlsx, 10 KB)
